# Molecular Diversity, Structure–Function Relationship, Mechanism of Action, and Transformative Potential of Black Soldier Fly Antimicrobial Peptides Against Multidrug-Resistant Pathogens

**DOI:** 10.3390/cimb48010062

**Published:** 2026-01-05

**Authors:** Ru-Xi Yuan, Xiao-Yang Ma, Yang Lv, Hong-Bin Si

**Affiliations:** 1 College of Animal Science and Technology, Guangxi Key Laboratory of Animal Breeding, Disease Control and Prevention, Guangxi Grass Station, Guangxi University, Nanning 530004, China; xi-2008@163.com (R.-X.Y.); 2 College of Animal Science and Technology, Qingdao Agricultural University, Qingdao 266109, China

**Keywords:** black soldier fly, antimicrobial peptides, antibiotic resistance, mechanism of action, analytical diversity, artificial intelligence, multidrug resistance

## Abstract

This review aims to systematically synthesize recent research advances on the antimicrobial peptides (AMPs) derived from the black soldier fly (*Hermetia illucens*). Against the backdrop of the escalating global crisis of antimicrobial resistance (AMR), AMPs have emerged as pivotal candidates to replace conventional antibiotics. As a unique saprophagous insect, *H. illucens* has evolved a robust and efficient innate immune system to thrive in its pathogen-rich environment. The AMPs it produces demonstrate remarkable broad-spectrum activity, high stability, and a low propensity for inducing resistance. Based on cutting-edge research available up to 2025, this article will provide an in-depth exploration of the astounding molecular diversity of *H. illucens* AMPs, their key structure–function relationships, and their multifaceted mechanisms of action, ranging from membrane disruption to immunomodulation. It will also highlight engineering strategies driven by artificial intelligence (AI). Finally, the review will assess the significant translational potential of these AMPs in combating multidrug-resistant bacteria, analyzing the current status of research in animal models, the challenges for industrial production, and viable future development pathways. The goal is to provide a solid theoretical foundation and forward-looking perspective to facilitate the translation of this valuable biological resource from basic research to clinical and agricultural applications.

## 1. Introduction

### 1.1. The Grave Challenge of AMR

AMR has escalated from a potential medical challenge to a present-day crisis, devastatingly impacting global public health security, economic stability, and sustainable animal husbandry [1]. Worldwide in 2019, bacterial AMR was linked to 4.95 million deaths, 1.27 million of which were directly attributable to it. Projections indicate that by 2050, AMR could directly cause up to 10 million annual deaths, with the most severe impacts anticipated in Asia and Africa [2].

The situation on the clinical frontline is particularly alarming. A 2024 global multicenter surveillance report indicates that the prevalence of multidrug-resistant pathogens has reached a critically high level [3]. Among these, carbapenem-resistant Enterobacteriaceae are of grave concern. Carbapenems are often considered a “last line of defense” antibiotic, yet the resistance rate among clinical CRE isolates was reported at 37% [3]. Another classic “superbug,” methicillin-resistant Staphylococcus aureus, was detected at a rate of 29% [2]. Furthermore, Acinetobacter baumannii, a major cause of opportunistic infections, exhibited an even higher multidrug resistance rate of 41% [4]. The widespread dissemination of these superbugs has resulted in a treatment failure rate exceeding 60% for severe nosocomial infections, substantially elevating both patient mortality risks and healthcare burdens.

The spread of AMR is not confined to healthcare settings. The long-term and large-scale use of antibiotics in animal husbandry for disease prevention and growth promotion has created an ideal breeding ground for the selection, evolution, and dissemination of resistant strains. For instance, in intensive farming environments, the resistance rate of Salmonella to low-cost tetracycline antibiotics has reached a prevalent high of 78% [5]. More alarmingly, bacteria carrying potent resistance genes can efficiently undergo horizontal transfer between animals and humans via complex pathways, including the environment and the food chain, posing a substantial risk of cross-contamination to global health [6]. A landmark study published in Science Advances in 2025 confirmed that a novel broad-spectrum β-lactamase resistance gene has been widely detected in urban wastewater samples across 23 countries, signaling that the AMR crisis is on the verge of becoming a globally uncontained phenomenon. ESKAPE pathogens, notably Klebsiella pneumoniae, Pseudomonas aeruginosa, and Acinetobacter baumannii [4], exhibit extensive resistance to nearly all existing classes of antibiotics, progressively depleting viable clinical treatment options and confronting physicians with the dire predicament of having no effective antibiotics left [3].

### 1.2. The Rise of AMPs as an Alternative Strategy

Facing the critical challenge of a “growing gap”—where the development of traditional antibiotics (averaging 10–15 years per new drug) lags significantly behind the evolution of bacterial resistance (which can emerge in as little as 2–3 years) [1], there is an urgent need for academia and industry to transcend conventional paradigms and develop entirely novel anti-infection strategies. AMPs, serving as core effector molecules of the innate immune system in multicellular organisms, are demonstrating considerable application potential due to their unique mechanisms of action and superior biological properties. They are regarded as one of the most promising candidates for the post-antibiotic era [2].

Unlike traditional antibiotics, which typically function as small-molecule inhibitors targeting specific bacterial enzymes or metabolic pathways, most AMPs exert their effects through a more direct and physically disruptive mechanism [7]. These short peptides generally possess a net positive charge and adopt amphipathic secondary structures. This characteristic allows them to preferentially target and adsorb to the negatively charged surfaces of microbial cell membranes via electrostatic attraction, while demonstrating relatively low affinity for the electrically neutral membranes of mammalian cells, thereby achieving selective pathogen killing. Following adsorption, AMPs disrupt membrane integrity through various models [8]. For instance, they may insert into the membrane akin to barrel staves, forming transmembrane pores according to the “barrel-stave model”; alternatively, they may induce lipid monolayers to curve, forming “toroidal-pore model” channels; or, at high concentrations, they may coat the membrane surface in a “carpet-like model”, ultimately solubilizing it. This primary mechanism—disrupting the physical structure of the cell membrane—is multi-targeted and non-specific, making it exceedingly difficult for bacteria to develop effective resistance through single or limited mutations. The estimated frequency for bacteria developing resistance to AMPs is only 1/100 to 1/1000 of that observed for traditional antibiotics.

The advantages of AMPs extend even further. They often exhibit remarkably broad-spectrum activity, demonstrating efficacy against Gram-positive bacteria, Gram-negative bacteria, fungi, enveloped viruses, and even parasites [9]. Moreover, many AMPs possess potent immunomodulatory functions [10]. For example, human β-defensin 3 not only exerts direct antimicrobial activity but also acts as a chemoattractant, recruiting dendritic cells and memory T cells to infection sites, thereby bridging innate and adaptive immune responses. This dual functionality combines direct pathogen elimination with indirect enhancement of host defenses. These exceptional properties have greatly accelerated their clinical translation [11]. As of the end of 2024, 86 peptide-based antimicrobial drugs had gained approval worldwide, 17 of which are specifically indicated for treating multidrug-resistant infections, with over 592 candidate peptides in various preclinical or clinical trial stages, highlighting their substantial market potential.

### 1.3. Hermetia illucens: A Unique Source of Antimicrobial Peptides

Despite the considerable promise of AMPs, their broad-scale application faces several challenges. These include the susceptibility of natural AMPs to proteolytic degradation in vivo, low bioavailability, high production costs from chemical synthesis or recombinant expression, and potential drawbacks such as cytotoxicity or hemolytic activity observed with some peptides at elevated concentrations [12]. This context has propelled global interest in discovering and screening novel AMPs from organisms in specific niches, focusing on identifying candidates with novel structures, enhanced activity, and superior stability.

**Figure 1:**
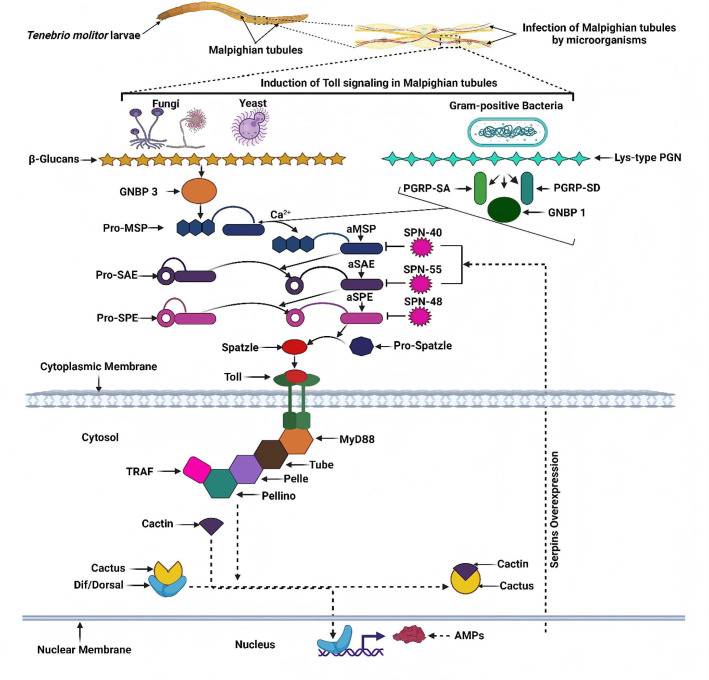
Molecular process of the Toll signaling pathway in *Hermetia illucens* (reprinted with permission from ref. [14], copyright 2024, *Entomological Research*). The diagram illustrates that the Spätzle protein processing initiates a serine protease cascade. Following downstream signal activation, this cascade promotes the nuclear translocation of the transcription factors Dif and Dorsal, ultimately regulating the transcription of antimicrobial peptide genes. The process is also modulated by a protease inhibitor complex.

The black soldier fly, *Hermetia illucens*, a globally distributed saprophagous insect, has garnered considerable interest due to its unique biological traits. The larvae thrive in decaying organic matter, environments laden with high concentrations of pathogenic microbes (total bacterial counts can reach up to 10^6^ CFU/g), yet they exhibit remarkable disease resistance. This resilience directly indicates the evolution of a highly efficient and potent innate immune system [13]. Upon challenge with pathogen-associated molecular patterns such as bacterial cell wall components, key signaling pathways in *H. illucens*—specifically the Toll and IMD (Immune Deficiency) pathways—are synergistically activated. This rapid activation triggers the massive synthesis and secretion of diverse AMPs from tissues like the fat body and hemocytes into the hemolymph (Figure 1), establishing a robust chemical defense barrier [13]. This efficient inducible expression system presents a promising strategy for the large-scale production of AMPs. For example, studies report that LPS stimulation can lead to a dramatic upregulation of Defensin gene expression in *H. illucens* by as much as 12.7-fold.

More importantly, to adapt to the complex acidic and protease-rich environment of its digestive tract, AMPs derived from *H. illucens* typically exhibit exceptional physicochemical stability. This remarkable stability not only suggests the feasibility of developing oral formulations but also provides a significant advantage for its application in complex in vitro environments, such as in feed additives or wound dressings.

As of 2025, research on *H. illucens* AMPs has progressed from initial isolation and identification to a new phase characterized by systematic exploration based on genomics and proteomics [13], as well as precision design and optimization leveraging artificial intelligence (AI). However, existing reviews either fail to encompass the latest molecular discoveries or provide insufficient discussion on cutting-edge areas such as structural biology evidence and AI-driven design. Therefore, this review will synthesize high-impact research published between 2020 and 2025 to comprehensively delineate the research landscape of *H. illucens* AMPs, aiming to provide a solid theoretical foundation and technical reference to accelerate their translation from basic research to industrial application.

## 2. Molecular Diversity and Identification of Antimicrobial Peptides from *Hermetia illucens*

*Hermetia illucens* is recognized as one of the insects possessing the most abundant and diverse repertoire of AMP genes identified to date. Facilitated by the widespread application of high-throughput sequencing and advanced bioinformatics tools, research as of 2025 has identified at least 57 AMPs with confirmed or potential bioactivity from their hemolymph, fat body, and gut. These AMPs, which can be classified into at least eight distinct families, exhibit remarkable molecular diversity. This diversity provides the host with a sophisticated, synergistic, and highly efficient “molecular arsenal,” enabling a tailored defense against invasions by various pathogens with differing challenge levels.

### 2.1. Major AMP Families and Their Characteristics

*Hermetia illucens* possesses a diverse repertoire of AMP families, which include several classic categories central to insect immunity (Table 1). Each category is characterized by distinct structural and functional properties.

- Defensins: This group comprises small, cysteine-rich cationic peptides that are crucial to the fly’s immune defense. Their structure is stabilized by 3–4 intramolecular disulfide bonds, forming a compact cysteine-stabilized αβ motif that consists of an α-helix and two antiparallel β-sheets. This configuration confers remarkable resistance to heat and proteolytic degradation. Several defensin-like peptides, such as DLP2, DLP4, and DLP5, have been identified in *H. illucens*. They exhibit potent activity against Gram-positive bacteria, including Staphylococcus aureus [15], primarily by interacting with specific membrane lipids to disrupt membrane integrity [7]. For example, DLP4 shows significant efficacy against various Gram-positive bacteria, with minimum inhibitory concentration values in the micromolar range.
- Cecropins: Among the most extensively studied AMPs in *H. illucens*, cecropins demonstrate particularly potent activity. Unlike defensins, they are linear, cysteine-free peptides that adopt an amphipathic α-helical conformation [16]. Members like Cecropin Z1 display broad-spectrum antimicrobial activity, effective against both Gram-positive and Gram-negative bacteria, with a pronounced effect on the latter. Their mechanism involves the “carpet model,” where the amphipathic helix lies parallel to the membrane surface and, at a critical concentration, disrupts it through a detergent-like effect, causing content leakage [10]. This family is considered a major contributor to the overall antimicrobial activity observed in *H. illucens*.
- Attacins: These are relatively large (~20 kDa), glycine-rich peptides, with identified members including HI-attacin and Hill_BB_C10074 [13]. They function mainly against Gram-negative bacteria by inhibiting the synthesis of outer membrane proteins, thereby increasing membrane permeability. While their direct bactericidal activity is limited, attacins exhibit strong synergy with other AMPs, such as cecropins and lysozymes. They are thought to facilitate the action of these co-acting AMPs by compromising the outer membrane barrier.
- Diptericins: Similar to attacins, diptericins are glycine-rich peptides active against Gram-negative bacteria [17]. Their proposed mechanism also involves disrupting bacterial membrane function, although the molecular details require further elucidation.
- Lysozymes: These are enzymes that cleave the β-1,4-glycosidic linkages in peptidoglycan, the primary structural polymer of bacterial cell walls. Several lysozyme genes have been identified in *H. illucens*.

**Table 1:** Representative antimicrobial peptides identified from *Hermetia illucens* and their reported antimicrobial spectra [18].

Serial Number	AMP Family	AMP Example	Activity Against
1	Attacin	HI-attacin	*MRSAb* *E. coli* KCCM 11,234
2	Cecropin	CLPI 1	*P. aeruginosa* KCCM 11,328 *E. coli* KCCM 11,234 *E. aerogenes* KCCM 12,177
		Trx-somoxynZHla	*S. aureus* *E. coli*
3	Defensin	DLP 2	*S. aureus* CICC 546 *S. aureus* ATCC 6538 *S. aureus* ATCC 25,923 *S. aureus* ATCC 43,300 *S. suis* CVCC 606 *L. ivanovii* ATCC 19,119
		DLP 3	*S. aureus* KCCM 12,256 *S. aureus* KCCM 40,881 *MRSAb* *S. epidermis* KCCM 25,494 *E. coli* KCCM 11,234 *P. aeruginosa* KCCM 11,328
		DLP 4	*S. aureus* CICC 546 *S. aureus* ATCC 6538 *S. aureus* ATCC 25,923 *S. aureus* ATCC 43,300 *S. suis* CVCC 606 *L. ivanovii* ATCC 19,119
			*S. aureus* KCCM 12,256 *S. aureus* KCCM 40,881 *MRSAb* *S. epidernidis* KCCM 35,494 *B. subtilis* KCCM 11,316
			*S. snis* CVCC 3928 *S. epidernis* ATCC 12,228 *S. aureus* CVCC 546 *S. pnelumoniae* CVCC 2350
		Hidefensin-1	*E. coli*
		Hill BB_C 6571 Hill_BB_C 7985 Hill_BB_C 16,634 Hill_BB_C 46,948	*E. coli*
		ID 13	*S. snis* CVCC 3928 *S. aureus* CVCC 546 *S. pneumoniae* CVCC 2350 *S. epidernis* ATCC 12,228
4	Diptericin	Hidiptericin-1	*S. pneumoniae* *E. coli*
5	IATP	HiCG 13,551	*S. aureus* *S. pneumoniae* *E. coli*

- Other Families: In addition to the major families described above, bioinformatic predictions and transcriptome analyses have also identified homologs of other AMP families in *Hermetia illucens*, including Sarcotoxins, Stomoxysins, and Coleoptericins [19].
- It should be pointed out that the data on the antibacterial activity of *Hermetia illucens* antimicrobial peptides presented in Table 1 and cited in this paper are derived from multiple independent studies, which exhibit certain differences in experimental methodologies. For instance, the endpoint indicators for activity evaluation may include the minimum inhibitory concentration, minimum bactericidal concentration, or diameter of the inhibition zone; additionally, the culture media used, bacterial inoculum size, peptide purity and concentration may also vary among these studies. Therefore, the “Activity against” summarized in Table 1 should be regarded primarily as a qualitative indicator of the activity range rather than standardized data that can be directly subjected to precise quantitative comparison. Future research needs to establish unified standards for in vitro pharmacodynamic evaluation to enable more reliable comparison of the efficacy of different AMPs.

### 2.2. Systematic Mining Based on Omics Technologies

In recent years, research strategies leveraging high-throughput omics technologies have significantly accelerated the discovery of AMPs in the black soldier fly, *Hermetia illucens*.

- Transcriptomics: This approach is currently the most prominent method for mining novel AMPs. Researchers perform RNA sequencing on *H. illucens* larvae, comparing samples that are either uninduced or induced with bacteria or pathogen-associated molecular patterns. This allows for the identification of differentially expressed genes [20]. Genes that are significantly upregulated post-induction, particularly those encoding small, secretory, and cationic peptides, become primary candidates for novel AMPs. This strategy has led to the discovery of not only numerous homologs of known AMPs but also entirely novel peptides like DLP-5, which lack known homologous sequences.
- Proteomics: This technique provides analysis at the protein level, offering direct evidence for the actual existence and post-translational modifications of AMPs. Typically, hemolymph is collected from induced larvae, followed by separation and purification using high-performance liquid chromatography. The resulting fractions are then identified using mass spectrometry techniques, such as MALDI-TOF/TOF MS [21]. This “top-down” methodology not only validates peptides predicted by transcriptomics but also discovers mature, active peptides. These mature forms result from direct gene translation followed by modifications such as cleavage, acetylation, or amidation, which are crucial for understanding the true functional activity of AMPs.
- Bioinformatics and Database Utilization: The efficient interpretation of omics data relies heavily on powerful bioinformatics tools and public databases. Researchers utilize databases like NCBI and UniProt for sequence alignment and homology analysis. More importantly, specialized AMP databases, such as the Antimicrobial Peptide Database, the Collection of Antimicrobial Peptides, and the Database of Antimicrobial Peptides, are indispensable [10]. These resources not only catalog sequence and structural information for thousands of known AMPs but also incorporate various prediction algorithms. These algorithms can screen massive transcriptomic or genomic datasets to predict and prioritize potential AMP candidates based on parameters like amino acid composition, net charge, hydrophobicity, and secondary structure propensity, thereby greatly enhancing discovery efficiency.

By integrating these omics technologies, our understanding of *H. illucens* AMPs has evolved beyond individual peptides to the construction of a comprehensive, multi-layered molecular map. This foundational work paves the way for subsequent functional studies and development applications.

## 3. Structure–Function Relationship and Mechanism of Action

The potent antimicrobial activity of black *H. illucens* AMPs is rooted in their unique molecular structures. Understanding the structure–function relationship and elucidating their multifaceted mechanisms of action are crucial for the rational design and optimization of next-generation antimicrobial agents.

### 3.1. Critical Domains and Antimicrobial Activity

The bioactivity of AMPs is governed by both the physicochemical properties inherent in their primary amino acid sequence and the three-dimensional structures into which they fold [10].

- Cationicity: Most AMPs from the black soldier fly exhibit a high isoelectric point (pI), conferring a net positive charge under physiological pH conditions [22]. This property is determined by an abundance of basic amino acid residues, such as arginine and lysine, in their sequences [16]. This cationic nature is the initial step for target selectivity, enabling AMPs to preferentially adsorb onto the generally negatively charged bacterial membrane surfaces via electrostatic attraction, while interacting only weakly with the neutral membranes of mammalian cells [23].
- Amphipathicity: This is the key structural feature that allows AMPs to insert into and disrupt cell membranes [9]. In their active conformations, hydrophobic amino acid residues and hydrophilic/charged residues segregate to opposite faces of the structure, forming a distinct hydrophobic facet and a hydrophilic facet [7]. This amphipathic architecture enables the AMP, upon membrane binding, to stably insert its hydrophobic face into the lipid core of the membrane, thereby disrupting its ordered packing and ultimately increasing membrane permeability.

Specific Three-Dimensional Structural Motifs:

- CSαβ Motif: For the defensin family, activity is guaranteed by the Cysteine-Stabilized α-helix/β-sheet motif, which is locked in place by multiple disulfide bonds. This rigid structure not only confers resistance to proteases and extreme pH/temperature but also precisely orients key amino acid residues, allowing the peptide to interact with specific receptors or lipid molecules on the bacterial membrane in an optimal configuration [23].
- α-Helix: For linear peptides like cecropins, the formation of a stable α-helix is a prerequisite for their activity. The helix length and the magnitude of its hydrophobic moment directly influence the depth of membrane insertion and the efficiency of membrane disruption [17].

### 3.2. Multidimensional Mechanism of Action

**Figure 2:**
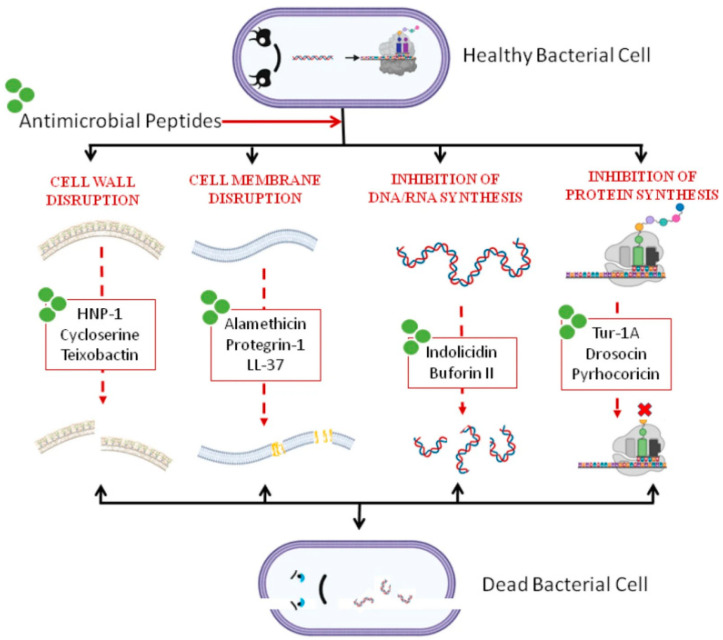
General mechanisms of action of antimicrobial peptides, relevant to *Hermetia illucens* AMPs (reprinted with permission from ref. [24], copyright 2024, *Archives of Microbiology*). The figure illustrates four broad mechanisms: Disruption of the bacterial cell wall, Disruption of the bacterial cell membrane, Inhibition of bacterial DNA/RNA synthesis, Inhibition of bacterial protein synthesis. Many AMPs from *H. illucens*, such as cecropins and defensins, are known or predicted to operate through mechanisms analogous to Disruption of the bacterial cell membrane and potentially Disruption of the bacterial cell wall, Inhibition of bacterial DNA/RNA synthesis, or Inhibition of bacterial protein synthesis.

Black soldier fly AMPs do not kill pathogens through a single pathway but rather exhibit a multidimensional and multi-level synergistic network (Figure 2).

- Membrane-Targeting Mechanisms: This represents the primary and most rapid bactericidal mechanism. As mentioned previously, AMPs can cause irreversible damage to the bacterial cell membrane within minutes via models such as the “barrel-stave”, “toroidal-pore”, or “carpet” models. This leads to the collapse of critical ion gradients and leakage of cellular contents [24]. This physical disruption is a major reason why bacteria struggle to develop resistance.
- Non-Membrane-Targeting Mechanisms: Recent studies have revealed that some AMPs, after traversing the cell membrane, can interact with crucial macromolecules inside the cell, dismantling vital bacterial processes from within. These intracellular targets include:
- Inhibition of Nucleic Acid Synthesis: Certain AMPs can bind to DNA or RNA, obstructing replication and transcription processes [24].
- Inhibition of Protein Synthesis: They may bind to ribosomes, interfering with the translation process.
- Inhibition of Enzymatic Activity: They target and inhibit essential enzymes involved in key pathways such as cell wall synthesis and energy metabolism.

This multi-target strategy, attacking from both inside and outside, further reduces the risk of resistance development [17].

**Figure 3:**
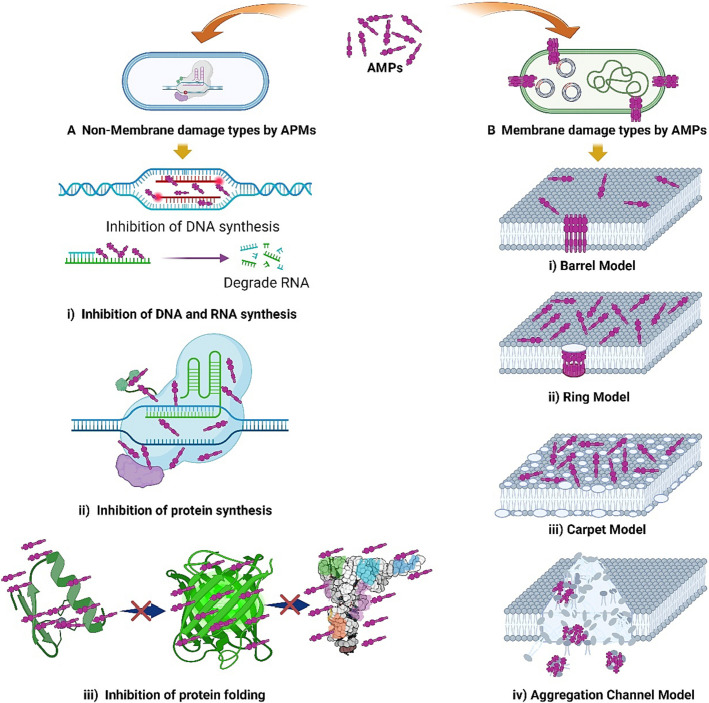
Schematic of *H. illucens* AMPs mechanisms (reprinted with permission from ref. [14], copyright 2024, *Entomological Research*). (**A**) Non-membrane-targeting mechanisms: (i) inhibit DNA/RNA synthesis, (ii) block protein synthesis, (iii) impair protein folding; (**B**) Membrane-damaging mechanisms: (i) Barrel Model, (ii) Ring Model, (iii) Carpet Model, (iv) Aggregation Channel Model. The membrane-disruptive actions (**B**) are particularly representative of the primary mode of action for many linear *H. illucens* AMPs like cecropins. The intracellular mechanisms (**A**) represent potential secondary or alternative targets for some insect-derived AMPs.

- Immunomodulatory Functions: AMPs are not only “killers” but also vital “messengers” (Figure 3). In in vitro cell experiments, HI-3, an antimicrobial peptide derived from *Hermetia illucens*, has been proven to exert a significant immunomodulatory effect on RAW264.7 murine macrophage cells and is capable of regulating the release of cytokines such as tumor necrosis factor-α and interleukin-6 [25]. These cell-based findings indicate that antimicrobial peptides derived from *Hermetia illucens* may not only directly eliminate pathogens but also indirectly enhance the anti-infection capacity by regulating the host’s inflammatory response [26].
- Synergistic Effects: A notable feature of the black soldier fly immune system is the coordinated expression of multiple AMPs [27]. For instance, attacins disrupt the outer membrane of Gram-negative bacteria, creating conditions for smaller yet more potent cecropins to penetrate and damage the inner membrane. The combined bactericidal effect of these two AMPs far exceeds the sum of their individual effects [28,29]. This natural synergy suggests that future drug development could focus on designing AMP-based “cocktail therapies” targeting different mechanisms to combat more stubborn multidrug-resistant infections [24,30].

## 4. Engineering Modification and Optimization of *Hermetia illucens*-Derived Antimicrobial Peptides: Advances and Future Prospects

Although antimicrobial peptides identified from natural *Hermetia illucens* exhibit remarkable antibacterial activity, their direct clinical translation is confronted with multiple challenges, including potency, stability, production cost and toxicity. Therefore, systematic engineering modification represents a key step to transform these peptides into therapeutic agents. Compared with model insects such as Drosophila melanogaster and Bombyx mori, the engineering research of *H. illucens* AMPs is still in the early stage but is developing rapidly. The revolutionary advances in artificial intelligence (AI) technology have provided brand-new methodologies and opportunities for leapfrog development in this field. This section will systematically summarize the traditional engineering exploration of *H. illucens* AMPs and conduct an in-depth analysis of the potential, pathways and core challenges of AI-driven rational design strategies.

### 4.1. Traditional Engineering Exploration of Hermetia illucens Antimicrobial Peptides

At present, engineering efforts targeting antimicrobial peptides derived from *Hermetia illucens* have mainly focused on their discovery, structural characterization and development for specific applications, which have laid a foundation for advanced molecular design.

#### 4.1.1. Identification and Structural Characterization of Core Functional Sequences

Accurate identification of functional sequences is the starting point for engineering modification [31]. Through pathogen induction and omics analysis, researchers have cloned and obtained a number of novel antimicrobial peptides from *Hermetia illucens*. Defensin peptide DFS1 was identified to possess a typical Knottin domain stabilized by three pairs of disulfide bonds, which serves as the structural basis for its membrane-permeabilizing activity [32]. Evolutionary analysis has indicated the functional conservation of this peptide. Such studies have provided precise template sequences and key functional domain information. In another study on cationic helical peptide HIMAMP1, alanine scanning was employed to clarify the decisive role of its hydrophobic face in mediating antibacterial activity [33], which has offered direct evidence for the design of minimal active fragments and site-directed mutagenesis.

#### 4.1.2. Functional Development for Large-Scale Production and Application

Translating laboratory discoveries into products constitutes a critical step in the engineering pipeline [34]. Research has focused on the feasibility of large-scale preparation; the response surface methodology was adopted to optimize the combined ultrasound-enzymatic extraction process, with peptide yield and minimum inhibitory concentration set as dual objectives to determine the optimal parameters [35]. This embodies the engineering mindset of transitioning from gram-scale preparation to industrial production. On the application front, the most promising direction lies in the development of alternatives to feed antibiotics. Studies have demonstrated that supplementing animal feed with a specific proportion of crude extracts of *Hermetia illucens* antimicrobial peptides can significantly improve intestinal health and enhance growth performance in animals [36]. Such efficacy-verification studies conducted in complex living organisms have accumulated valuable in vivo data for the future development of dosage forms for therapeutic applications.

### 4.2. AI-Driven Rational Design

The paradigm-shifting breakthroughs of artificial intelligence (AI) in the field of universal antimicrobial peptide (AMP) design have provided a clear technical roadmap for *Hermetia illucens* research. Cutting-edge studies are transitioning from a paradigm of passive screening to active generation and directed evolution, aiming to address the challenge of multi-objective optimal molecular design.

#### 4.2.1. From Virtual Screening to Intelligent Generation

The core advantage of next-generation generative AI lies in its ability to explore an almost infinite space of unknown sequences [37]. By learning to reconstruct data distributions from noise, diffusion models can controllably generate highly active sequences that satisfy constraints of specific physicochemical properties [38]. Another powerful paradigm is deep reinforcement learning. Models such as AMPainter frame molecular design as a sequence space exploration task, where the agent acquires rewards through interaction with the environment, thereby learning how to “evolve” optimal sequences. This approach enables both the directed evolution of known peptides and de novo design [39].

#### 4.2.2. AI-Enabled Strategies for *Hermetia illucens* Antimicrobial Peptides

By combining general-purpose AI tools with the current status of *Hermetia illucens* research, three specific technology integration pathways can be formulated.

First is intelligent directed evolution based on known templates. Using validated *H. illucens* antimicrobial peptides as “seeds”, deep reinforcement learning models are employed to perform multiple rounds of virtual iterative mutagenesis on them. The optimization objectives can be set to significantly reduce hemolytic toxicity by adjusting amino acid composition while maintaining or enhancing activity against target bacteria [32,40].

Second is de novo generation guided by structural priors. The key structural features of *H. illucens* defensins are incorporated into generative models as hard constraints. In this way, AI can act as a “molecular architect” to batch-design novel variants that belong to the “ *H. illucens* defensin family” in terms of topological structure but feature entirely new sequences and potentially superior functions [41].

Third is cross-species knowledge transfer and functional hybridization. Large language models (LLMs) pre-trained on extensive general protein databases encapsulate rich knowledge of protein sequence grammar. Through fine-tuning or prompt engineering, the models can be guided to generate or optimize hybrid peptide sequences that simultaneously possess the structural stability of *H. illucens* peptides and the efficient membrane-penetrating ability of peptides from other species [42].

#### 4.2.3. Key Challenges and Future Opportunities

While embracing the potential of AI, we must clearly recognize the fundamental bottlenecks in its application to the *Hermetia illucens* system. These challenges are precisely the positive opportunities that guide future research and drive the development of this field.

The primary challenge lies in the inherent contradiction between data scarcity and model generalization. The effectiveness of AI models fundamentally depends on large-scale, high-quality training datasets, which directly conflicts with the current status of severely insufficient validated sequences and activity data in *H. illucens* AMPs research. This “few-shot learning” dilemma can easily lead to model overfitting and restrict design innovation due to dataset bias. Therefore, constructing an open and shared *H. illucens*-specific multi-omics database represents the most fundamental and urgent opportunity to break through the current bottleneck [43].

Second, there exists a significant “confidence gap” between prediction and validation. AI may generate a large number of “false-positive” sequences with theoretically perfect indicators, which may fail in real physiological environments due to misfolding or rapid degradation. The core opportunity to narrow this gap is to establish an efficient, automated “design–build–test–learn” closed-loop experimental platform. All experimental results should be standardized and fed back to the model, enabling it to learn from errors, iterate and evolve, and thus make predictive models increasingly consistent with the true complexity of biology [34].

Finally, the complexity of multi-objective collaborative optimization constitutes another major challenge. An ideal antimicrobial peptide needs to balance multiple properties, including activity, stability, safety and producibility, among which inherent conflicts often exist. Targeting the specific folding requirements of *Hermetia illucens* peptides, a significant future opportunity lies in developing next-generation integrated AI frameworks capable of simultaneously addressing multi-objective Pareto frontier optimization and integrating early developability prediction, thereby enabling the intelligent design leap from “bioactive molecules” to “drug candidates” [44].

### 4.3. Conclusions and Future Perspectives

AI deeply into the engineering of *H. illucens* AMPs constitutes a systematic project that requires close collaboration across multiple disciplines. The challenges identified in this paper—ranging from data infrastructure construction, validation of predictive authenticity to multi-attribute intelligent optimization—clearly delineate a strategic roadmap for future research. By co-constructing and sharing databases, developing algorithms suitable for few-shot learning, and establishing high-throughput verification platforms, these challenges will be transformed into a powerful driving force for innovation. Ultimately, a complete innovation chain integrating AI-driven rational design, automated experimental validation, and synthetic biology-based intelligent manufacturing is expected to turn *Hermetia illucens* into an intelligent and novel source of biopharmaceuticals to address the global crisis of antibiotic resistance. Future research aims not only to create superior next-generation *H. illucens* AMPs but also to establish a scalable paradigm for the intensive exploitation of insect-derived bioactive molecules.

## 5. Translational Potential Against Multidrug-Resistant Bacteria

Due to their unique mechanisms of action and broad-spectrum activity, *H. illucens* AMPs demonstrate significant translational potential in addressing the growing challenge of multidrug-resistant bacteria.

### 5.1. Evaluation of Antimicrobial Activity In Vitro

Numerous in vitro studies have confirmed that various AMPs isolated from or recombinantly expressed in the black soldier fly exhibit significant inhibitory effects against clinically important pathogens.

- Broad-Spectrum Activity: Research has demonstrated that black soldier fly crude extracts or purified AMPs can inhibit a range of Gram-positive bacteria and Gram-negative bacteria, as well as certain fungi [28].
- Activity Against MDR Pathogens: More importantly, these AMPs remain effective against many multidrug-resistant strains [23]. For instance, black soldier fly-derived cecropins have been shown to be active against Gram-negative pathogens, including MDR strains. Defensins from this insect, such as DLP2 and DLP4, display potent antimicrobial activity against various Gram-positive bacteria, with minimum inhibitory concentration values reaching the low micromolar range in some cases [16].
- Current Status and Challenges: Despite the encouraging evidence, as of 2025, published studies providing systematic MIC and minimum bactericidal concentration data for purified, single black soldier fly AMPs against internationally recognized “superbugs”—particularly MDR clinical isolates of the ESKAPE pathogens—remain relatively scarce (query result for 2024–2025 MIC data was sparse) [24]. Future research urgently needs to fill this gap by generating more comprehensive and clinically relevant in vitro susceptibility data.

### 5.2. Efficacy and Safety in Animal Models

Translating in vitro activity into in vivo efficacy is a critical step in the development of AMPs as drugs. However, research on the direct efficacy of black soldier fly AMPs against MDR pathogens in live animal infection models remains in its early stages.

- Prospects for Efficacy Studies: Future research should focus on establishing standardized animal infection models. These should include models for skin and soft tissue infections caused by MRSA [45], intra-abdominal infection/sepsis models induced by CRE [46], and pneumonia models mediated by multidrug-resistant Acinetobacter baumannii [47]. Within these models, the efficacy of black soldier fly AMPs should be evaluated via local or systemic administration, specifically assessing their capacity to reduce bacterial load, improve animal survival rates, and ameliorate inflammatory damage.
- Safety Assessment: Safety is a decisive factor determining clinical translation. In vitro safety assessments primarily include hemolysis assays [48] and cytotoxicity tests [49]. Multiple preliminary studies indicate that natural black soldier fly AMPs typically exhibit good selectivity, meaning they demonstrate low toxicity towards mammalian cells at effective bactericidal concentrations [50,51]. However, more comprehensive in vivo toxicological studies—including assessments of acute toxicity, long-term toxicity, and immunogenicity—are mandatory for future regulatory submissions [52]. Currently, detailed toxicological data for black soldier fly AMPs in animal models during the 2024–2025 period is similarly scarce.

### 5.3. Challenges and Pathways for Commercialization

Translating black soldier fly AMPs from the laboratory to the market necessitates overcoming a series of technical and economic challenges.

#### 5.3.1. Challenges

1. Large-Scale, Low-Cost Production: Direct extraction from insects yields very low quantities and is costly [53]. Chemical solid-phase synthesis is only suitable for short peptides, with costs increasing exponentially with peptide length [54]. Mainstream recombinant expression systems often face issues such as low yield, susceptibility to degradation by host proteases, or inclusion body formation when expressing cationic, low molecular weight AMPs [27].
2. In Vivo Stability and Delivery: Upon entering the systemic circulation, AMPs are susceptible to rapid degradation by proteases, resulting in a short half-life that limits their efficacy for systemic administration [55].
3. Regulatory Approval: As a novel class of biotherapeutics, the evaluation standards for the pharmacokinetics [16], pharmacodynamics [55], and safety profile of AMPs are still being refined, and their regulatory pathway is less clearly defined compared to traditional antibiotics [56].

#### 5.3.2. Development Pathways and Solutions

- Optimizing Production Systems: Exploring more suitable expression hosts, such as insect cell expression systems [28], may be more conducive to correct folding and post-translational modifications. Concurrently, genetic engineering of the black soldier fly itself could create “cell factories” that over-secrete specific AMPs. Leveraging its native inducible expression machinery and optimizing induction conditions also represent effective strategies for enhancing yield.
- Developing Advanced Delivery Systems: Encapsulating AMPs within nanoparticles (e.g., liposomes [57], polymeric nanoparticles [58]) or hydrogels [58] can effectively protect them from proteolytic degradation, enabling targeted release and sustained pharmacokinetics [59].
- Diversified Application Scenarios: Beyond their use as systemic therapeutics for severe MDR infections, black soldier fly AMPs hold significant promise in other fields. Potential applications include their development into topical formulations for treating burns and skin infections [53,60]; their use as coating materials for medical devices to prevent biofilm formation [28,61]; and their application as feed additives in livestock and aquaculture industries to replace prophylactic antibiotics [62], promote animal health [22], and help curb the emergence of AMR at its source.

## 6. Conclusions

In 2025, amidst the escalating global crisis of AMR, the development of novel antibacterial agents has become an urgent priority for safeguarding public health worldwide. This article systematically demonstrates that the *Hermetia illucens*, serving as a natural repository of bioactive molecules, produces AMPs, which represent a highly promising candidate arsenal against multidrug-resistant pathogens. This potential is attributed to their remarkable molecular diversity, unique physical bactericidal mechanisms, exceptional physicochemical stability, and potential immunomodulatory functions.

Research indicates that the black soldier fly possesses a complex AMP system encompassing multiple families, including defensins, cecropins, and attacins. The application of high-throughput technologies such as genomics and proteomics is continuously unveiling the full scope of its AMP repertoire. More excitingly, AI-driven rational design and optimization strategies are propelling AMP research and development into an unprecedented fast lane. Utilizing generative models and multi-objective optimization algorithms holds the promise of creating “super antimicrobial peptides” with enhanced potency, reduced toxicity, and superior stability.

However, translating the immense potential of black soldier fly AMPs into tangible clinical or commercial products remains a long-term endeavor. Current research bottlenecks primarily include: (1) a lack of systematic in vitro susceptibility data against clinically critical MDR pathogens, particularly ESKAPE strains; (2) a severe scarcity of in vivo pharmacodynamic efficacy and safety validation data within standardized animal infection models; and (3) the immaturity of large-scale, cost-effective production processes.

Prospects: Looking ahead to the future, we propose the following key research directions:

- Deepening Fundamental Research: Systematically identify all AMP families within the black soldier fly and employ structural biology techniques to elucidate the atomic-level details of the interactions between key AMPs and bacterial membranes or intracellular targets.
- Strengthening Translational Research: Conduct large-scale in vitro screening to establish activity profiles against a diverse panel of MDR clinical isolates, and accelerate the evaluation of pharmacodynamics and toxicology in animal models such as murine sepsis, pneumonia, and skin infection models.
- Focusing on AI and Engineering: Continue leveraging AI generative models to design novel AMP sequences with enhanced drug-like properties. Integrate chemical modifications and nanodelivery technologies to address the challenge of poor in vivo stability.
- Expanding Application Dimensions: While vigorously promoting their development as human prescription drugs, actively explore their applications in veterinary medicine, functional feed additives, and medical device coatings. This aims to realize their multidimensional value within the “One Health” framework.

In summary, research on black soldier fly antimicrobial peptides stands at a crossroads, brimming with both opportunities and challenges. Through interdisciplinary collaboration, we can confidently anticipate that these ancient defensive molecules, derived from nature, will provide novel and powerful solutions in humanity’s fight against “superbugs” in the near future.

## Data Availability

No new data were created or analyzed in this study. Data sharing is not applicable to this article.
