# Correction: Yuan et al. Molecular Diversity, Structure–Function Relationship, Mechanism of Action, and Transformative Potential of Black Soldier Fly Antimicrobial Peptides Against Multidrug-Resistant Pathogens. *Curr. Issues Mol. Biol.* 2026, *48*, 62

**DOI:** 10.3390/cimb48070685

**Published:** 2026-07-03

**Authors:** Ru-Xi Yuan, Xiao-Yang Ma, Yang Lv, Hong-Bin Si

**Affiliations:** 1 College of Animal Science and Technology, Guangxi Key Laboratory of Animal Breeding, Disease Control and Prevention, Guangxi Grass Station, Guangxi University, Nanning 530004, China; xi-2008@163.com (R.-X.Y.);; 2 College of Animal Science and Technology, Qingdao Agricultural University, Qingdao 266109, China

**Figure Legend**

In the original publication [1], there were two mistakes. First, the copyright permission statements were inadvertently omitted from the legends of Figures 1–3. The copyright permission statements were inadvertently omitted. Second, a literature attribution error occurred in Sections 1.3 and 2.1 (see below). The correct legend appears below and the incorrect sentence has been removed. 

**Correct Legend**

**Figure 1.** Molecular process of the Toll signaling pathway in *Hermetia illucens* (reprinted with permission from ref. [14], copyright 2024, *Entomological Research*). The diagram illustrates that the Spätzle protein processing initiates a serine protease cascade. Following downstream signal activation, this cascade promotes the nuclear translocation of the transcription factors Dif and Dorsal, ultimately regulating the transcription of antimicrobial peptide genes. The process is also modulated by a protease inhibitor complex.

**Figure 2.** General mechanisms of action of antimicrobial peptides, relevant to *Hermetia illucens* AMPs (reprinted with permission from ref. [24], copyright 2024, *Archives of Microbiology*). The figure illustrates four broad mechanisms: Disruption of the bacterial cell wall, Disruption of the bacterial cell membrane, Inhibition of bacterial DNA/RNA synthesis, Inhibition of bacterial protein synthesis. Many AMPs from *H. illucens*, such as cecropins and defensins, are known or predicted to operate through mechanisms analogous to Disruption of the bacterial cell membrane and potentially Disruption of the bacterial cell wall, Inhibition of bacterial DNA/RNA synthesis, or Inhibition of bacterial protein synthesis.

**Figure 3.** Schematic of *H. illucens* AMPs mechanisms (reprinted with permission from ref. [14], copyright 2024, *Entomological Research*). (**A**) Non-membrane-targeting mechanisms: (i) inhibit DNA/RNA synthesis, (ii) block protein synthesis, (iii) impair protein folding; (**B**) Membrane-damaging mechanisms: (i) Barrel Model, (ii) Ring Model, (iii) Carpet Model, (iv) Aggregation Channel Model. The membrane-disruptive actions (**B**) are particularly representative of the primary mode of action for many linear *H. illucens* AMPs like cecropins. The intracellular mechanisms (**A**) represent potential secondary or alternative targets for some insect-derived AMPs.

In the original publication, there was also a literature attribution error in Sections 1.3 and 2.1. The following sentences:

In Section 1.3: “It has been reported that Cecropin-α sourced from *H. illucens* retains 82% of its antimicrobial activity after heating at 100 °C for 30 min and maintains structural and functional stability across a broad pH range from 2.0 to 10.0 [15].” In Section 2.1: “They play a critical role in defense against Gram-positive bacteria by mediating the direct lysis of bacterial cells [15].” were incorrectly attributed to reference [15] due to a reference mismanagement errors. These sentences and their corresponding citation [15] have been removed from the article. As a result of corrections, the order of some references has been adjusted accordingly.

The authors state that the scientific conclusions are unaffected. This correction was approved by the Academic Editor. The original publication has also been updated.
